# Acetate free citrate-containing dialysate increase intact-PTH and BAP levels in the patients with low intact-PTH

**DOI:** 10.1186/1471-2369-14-18

**Published:** 2013-01-18

**Authors:** Takahiro Kuragano, Minoru Furuta, Mana Yahiro, Aritoshi Kida, Yoshinaga Otaki, Yukiko Hasuike, Akihide Matsumoto, Takeshi Nakanishi

**Affiliations:** 1 Department of Internal Medicine Division of kidney and Dialysis, Hyogo College of Medicine, Nishinomiya, Japan; 2 Seishoukai Aoi Hospital, Nishinomiya, Japan; 3 Takahiro Kuragano, 1-1, Mukogawa cho, 663-8501, Nishinomiya, Japan

**Keywords:** Acetate free citrate-containing dialysate, Low intact-parathyroid hormone, Bone alkaline phosphatase, Total calcium, Ionized calcium

## Abstract

**Background:**

Recently, acetate-free citrate containing dialysate (A(−)D) was developed. We have already reported about the significant effect of A(−)D on metabolic acidosis, anemia, and malnutrition in maintenance hemodialysis (MHD) patients. In this study, we compared the effect of A(−)D and acetate containing dialysate (A(+)D) on serum calcium and intact-parathyroid hormone (int-PTH) levels.

**Method:**

Single session study: Seventeen patients were treated with A(+)D in one session and also treated with A(−)D in another session. Serum levels of pH, HCO_3_^-^, total (t)-calcium, ionized (i)-calcium, and int-PTH were evaluated at the beginning and the end of each session. Cross over study: A total of 29 patients with MHD were treated with A(+)D for 4 months, switched to A(−)D for next 4 months, and returned to A(+)D for the final 4 months.

**Results:**

In single session study, serum i-calcium and t-calcium levels significantly increased, and int-PTH levels decreased after HD with A(+)D, whereas HD with A(−)D did not affect iCa and int-PTH. In cross over study, if all patients were analyzed, there was no significant difference in serum int-PTH or bone alkaline phosphatase (BAP) levels during each study period. In contrast, in the patients with low int-PTH (<60 pg/mL), serum levels of int-PTH and BAP were significantly increased during the A(−)D, without significant changes in serum t-calcium or i-calcium levels.

**Conclusion:**

A(−)D containing citrate could affect calcium and PTH levels, and, in 4 month period of crossover study, increased int-PTH levels pararelled with increasing BAP levels, exclusively in MHD patients with low int-PTH levels.

## Background

Newly developed acetate-free citrate containing dialysate (Carbostar^®^, Ajinomoto Pharmaceuticals Co., Ltd., Tokyo, Japan) is expected to be clinically effective in maintaining hemodynamic stability during hemodialysis (HD) sessions. We recently demonstrated in a cross-over study that HD with acetate-free citrate dialysate (A(−)D) could contribute to improved clinical status, including improved hemodynamic conditions during HD [1], metabolic acidosis, malnutrition, and ESA-hyporesponsive anemia, which were not normalized by HD with conventional dialysate [2]. A(−)D is characterized as total removal of acetate substituted by citrate as a buffer source (Table 1). Past studies have reported the impact of calcium concentrations in dialysates on bone mineral metabolism and int-PTH levels [3-5]. Furthermore, Karohl C et al. reported that calcium balance correlated with i-calcium gradients between plasma and dialysate and PTH levels [6]. From these reports, bone remodeling of MHD patients could be affected by calcium mass transfer during HD sessions. Because citrate is an effective chelator of ionized (i)-calcium, HD with A(−)D containing citrate may decrease serum i-calcium levels and affect int-PTH levels during HD sessions. Moreover, Lefebvre A et al. reported that optimal correction of metabolic acidosis by raising the dialysate base HCO_3_^-^ concentration affected PTH and osteocalcin levels and bone resorption surfaces in 21 MHD patients [7]. The purpose of this study is to determine the effect of varying the dialysate composition on the calcium and int-PTH metabolism in a single HD session and as part of an earlier cross-over study.

**Table 1:** Compositions of A(−)D and A(+)D

**Chemical**	**A(−)D**	**A(+)D**
Sodium (mEq/L)	140	140
Potassium (mEq/L)	2	2
Calcium (mEq/L)	3	3
Magnesium (mEq/L)	1	1
Chloride (mEq/L)	111	113
Bicarbonate (mEq/L)	35	25
Acetate (mEq/L)	0	10
Citrate (mEq/L)	2	0
Glucose (mEq/L)	150	100

A(−)D does not contain acetate as a buffering agent, and bicarbonate concentration of A(−)D is higher than that of A(+)D.

In this study, for clarifying the effect of A(−)D on calcium and int-PTH metabolism, we evaluated the changes of total (t)-calcium, i-calcium, and int-PTH in a single HD session and as part of previous cross-over study.

## Methods

### Patients

Seventeen patients (for the single session study) and 29 patients (for the cross-over study), who had received MHD for more than one year and who were in stable clinical condition, participated in the present study. Patients with chronic inflammatory diseases, malignant tumors, or severe hepatic or respiratory diseases were excluded from the study. Written informed consent was obtained from all patients. All subjects gave their informed consent in accordance with the requirements of the institutional committee on human research, and the committee approved the study protocol (Hyogo College of Medicine No. 436). The study was registered with the University Hospital Medical Information Network (UMIN) Clinical Trial Registry (UMIN number: 000000773).

The patients included in cross-over study were part of an earlier clinical trial designed to evaluate the effect of A(−)D on dialysis efficiency, nutritional status, inflammatory conditions, and response to ESA, as previously reported [2].

### Study design

#### Single session study

Seventeen MHD patients were treated with acetate-containing dialysate (A(+)D, AK-sorita^®^, Ajinomoto Pharmaceuticals Co., Ltd., Tokyo, Japan) in one session and with A(−)D in another session, without changing other dialysis conditions (dialysis membrane used, blood flow rate, dialysate flow rate, and dry weight). Blood samples were taken at the beginning and the end of each session. We measured pH and serum i-calcium, t-calcium, HCO_3_^-^, and int-PTH levels. The blood levels of pH, HCO3−, t-calcium, and i-calcium were measured immediately after sampling from patients. The remaining blood samples were stored at −30°C until the BAP and int-PTH were measured.

#### Cross-over study

Twenty-nine MHD patients were treated with A(+)D for 4 months (first A(+)D period), were switched to A(−)D for the next 4 months (A(−)D period) and were returned to A(+)D for the final 4 months (second A(+)D period). Other parameters of HD therapy, including the dialysis membrane used, blood flow rate, dialysate flow rate, and anticoagulant agent (drug type and dosage), except for dialysate, was unchanged during the entire study period. Standard unfractioned heparin (not citrate) was administered as an anticoagulant, and the schedule of administration was not altered during the study for each patient.

At the beginning of the study, 25 patients were treated using a phosphate binder (calcium acetate: n=17; sevelamar: n=8), 13 patients were treated using an active vitamin D compound (calcitriol: n=2; alfacalcidol: n=4; maxacalcitol: n=7), and 2 patients were treated using calcimimetics. There were no significant differences in the doses of phosphate binder, active vitamin D compound, and calcimimetics during the study period. Blood samples were collected before and after the HD session at the beginning of the study, at each switch of dialysate and at the end of the study. We measured serum pH values and levels of t-calcium, i-calcium, HCO_3_^-^, int-PTH, and bone-specific alkaline phosphatase (BAP) at the same time. Serum levels of BAP and int-PTH were measured by chemiluminescence enzyme immunoassay. The blood levels of pH, HCO3−, t-calcium, and i-calcium were measured immediately after sampling from patients. The remaining blood samples were stored at −30°C until the BAP and int-PTH were measured.

### Statistical analysis

All values are presented as the mean ± SD. Differences between pre- and post-HD levels were analyzed using the paired Student’s t-test. The Mann–Whitney test was used for the analysis of the changes of several parameters during cross-over study. P values of <0.05 were considered statistically significant. Statistical analyses were performed with the Statistical Package for Social Science (SPSS), version 18.0 (SPSS Inc., Chicago, IL, USA).

## Results

### Single session study

#### Patients

Seventeen MHD patients (mean age: 62 years old; mean dialysis vintage: 4 years) were enrolled in the single session study (Table 2). All patients were treated with HD three times weekly for 3–5 hrs/session for an average of 3.6 ± 0.3 hours. The treatment time was unchanged for each patient during the study. The average blood flow rate was 187 ± 18 mL/min, and the dialysate flow rate was 500 mL/min. Polysulfone (PS), polyethersulfone (PES), and polymethylmethacrylate (PMMA) membranes were used for HD in 35%, 58%, and 7% of patients, respectively.

**Table 2:** Baseline clinical characteristics of MHD patients at the start of this study

	**Single session study**	**Long term study**
Number of patients	17	29
Age (yo)	62 ± 12	61 ± 4
Sex	Male; 11 Female; 6	Male; 16 Female; 13
Height (cm)	161 ± 7	157 ± 4
Weight (kg)	56 ± 10	58 ± 4
Etiology	DM; 9 non-DM; 8	DM; 15 non-DM; 14
Duration of dialysis (years)	4 ± 4	7 ± 2

*DM* diabetes. Data are the mean ± SD.

#### Metabolic acidosis

**Figure 1:**
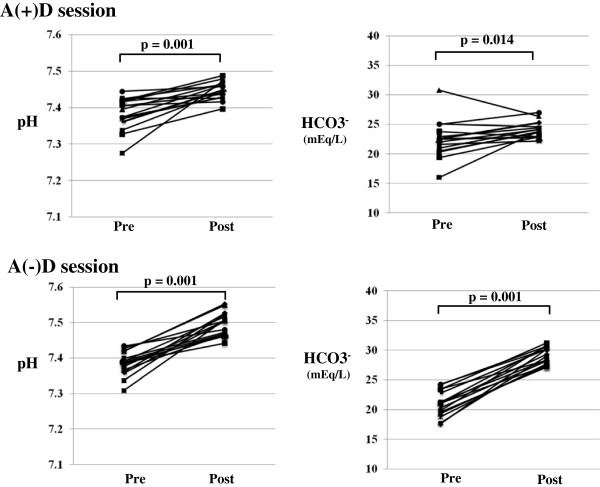
**Changes in pH and HCO**_**3**_^**- **^**after HD with A(−) and A(+)D in a single session.**

pH and HCO_3_^-^ significantly increased after HD sessions with both A(−)D and A(+)D, the increase of pH and HCO_3_^-^ with A(−)D (pH: from 7.38 ± 0.03 to 7.50 ± 0.03, p=0.001; HCO_3_^-^: from 21.0 ± 2.2 mEq/L to 29.2 ± 1.4, p=0.001) were significantly larger than A(+)D (pH: from 7.39 ± 0.04 to 7.45 ± 0.02, p=0.001; HCO_3_^-^: from 22.4 ± 3.1 to 24.3 ± 1.3, p=0.014) (Figure 1).

#### t-calcium and i-calcium

**Figure 2:**
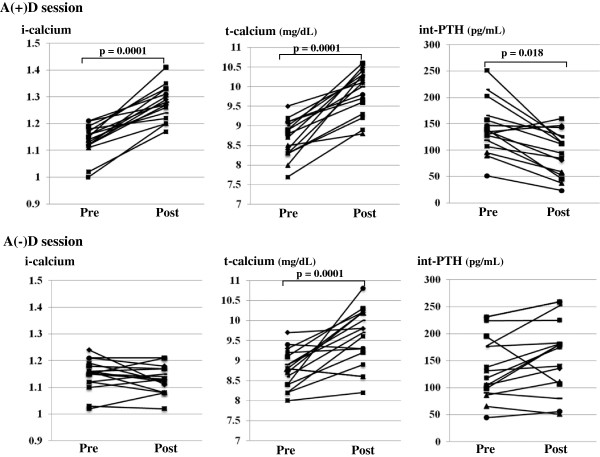
Changes in i-calcium, t-calcium, and int-PTH levels after HD with A(−)D and A(+)D in a single session.

After HD with A(+)D, both i-calcium (from 1.14 ± 0.06 mEq/L to 1.28 ± 0.06) and t-calcium (from 8.8 ± 0.48 mg/dL to 9.6 ± 0.68) significantly increased. On the other hand, after HD with A(−)D, although t-calcium significantly increased (from 8.7 ± 0.48 to 9.9 ± 0.56), there was no significant difference in i-calcium (from 1.14 ± 0.06 to 1.13 ± 0.05) between pre- and post-HD levels with A(−)D (Figure 2).

#### int-PTH levels

After HD sessions with A(+)D, serum levels of int-PTH were significantly decreased (from 149.9 ± 49.7 to 94.9 ± 42.0 pg/mL). In contrast, there was no significant difference in serum int-PTH between pre- HD (132.2 ± 54.8 pg/mL) and post-HD (154.4 ± 62.9 pg/mL) levels with A(−)D (Figure 2).

### Cross-over study

#### Patients

Twenty-nine MHD patients (mean age: 61 years old, mean dialysis vintage: 7 years) were enrolled in the cross-over study (Table 2). All patients were treated with HD three times weekly for 3–5 hrs/session for an average of 3.8 ± 0.5 hours. The treatment time was unchanged for each patient during the study. The average blood flow rate was 207 ± 18 mL/min, and the dialysate flow rate was 500 mL/min. Polysulfone (PS), polyethersulfone (PES), cellulose triacetate (CTA) and polymethylmethacrylate (PMMA) membranes were used for HD in 79%, 13%, 4% and 4% of patients, respectively. Ultrafiltration was subsequently adjusted according to each patient’s condition. The mean ultrafiltration per session was 1794 ± 560 mL in the first A(+)D period, 1944 ± 560 mL in the A(−)D period, and 1878 ± 565 mL in the second A(−)D period. However, no significant differences were noted in the total ultrafiltration in the three periods.

#### Metabolic acidosis

**Figure 3:**
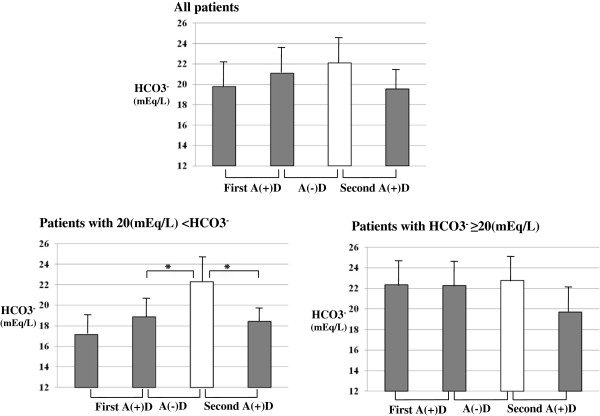
**Changes in HCO**_**3**_^**- **^**levels in all patients, patients with HCO**_**3**_^**- **^**≥ 20 mEq/L and patients with HCO**_**3**_^**- **^**< 20 mEq/L in the cross-over study. **Error bars are standard deviations. *P < 0.05.

In all patients, there were no significant differences in plasma HCO_3_^-^ levels during the 3 study periods. According to the pre-dialysis plasma HCO_3_^-^ levels at the beginning of the study, the patients were stratified into two groups ; HCO_3_^-^ levels ≥ 20 mEq/L (n=13) and < 20 mEq/L (n=16). In the patients with high plasma HCO_3_^-^ levels (≥ 20 mEq/L), there were no significant differences in plasma HCO_3_^-^ levels during the three study periods. Conversely, in the patients with low plasma HCO_3_^-^ levels (< 20 mEq/L), plasma HCO_3_^-^ levels were significantly (P < 0.05) increased from 17.8 ± 1.8 to 22 ± 3 mEq/L after switching from A(+)D to A(−)D. After switching back for the second period of A(+)D, they decreased to baseline levels (Figure 3).

#### t-calcium and i-calcium levels

**Figure 4:**
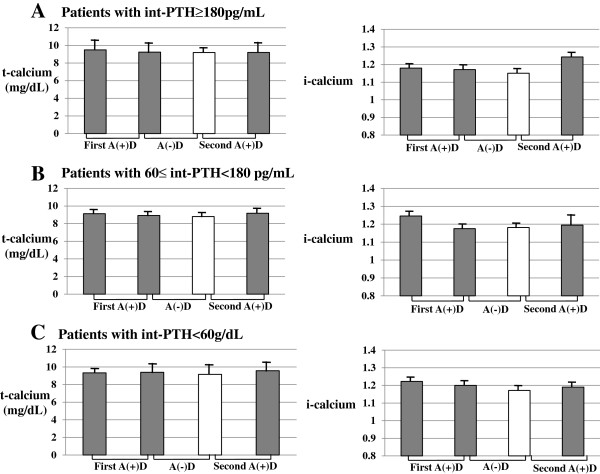
Changes in pre-dialysis t-calcium and i-calcium levels in the patients with int-PTH ≥ 180 pg/mL (A), 60–180 pg/mL (B), and int-PTH < 60 pg/mL (C) in the cross-over study.

In all patients, there were no significant changes in int-PTH levels during the study period. According to the serum int-PTH levels at the beginning of the study, the patients were divided into three groups: patients with int-PTH levels of < 60 pg/mL, those with 60–180 pg/mL, and those with ≥180 pg/mL. During the study period, there were no significant changes in serum t-calcium or i-calcium in any of groups (Figure 4A, B, and C).

#### int-PTH and BAP levels

**Figure 5:**
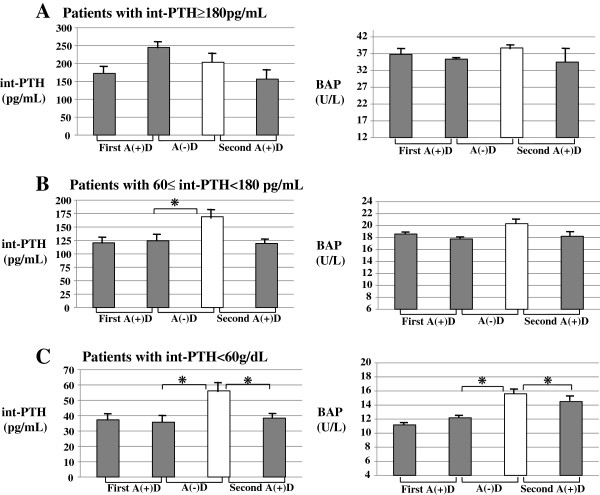
**Changes in pre-dialysis int-PTH and BAP levels in the patients with int-PTH ≥ 180 pg/mL (A), 60–180 pg/mL (B), and int-PTH < 60 pg/mL (C) during the study period. **int-PTH: intact parathyroid hormone; BAP: bone-specific alkaline phosphatase. Error bars are standard deviations. *P < 0.05.

In the patients with int-PTH ≥180 pg/mL, there were no significant changes in int-PTH levels during the study period (Figure 5A), whereas in the patients with int-PTH < 60 pg/mL and 60≤ int-PTH <180 pg/mL, especially in the patients with int-PTH < 60 pg/mL, int-PTH levels increased significantly (*P* < 0.05) after the switch from A(+)D to A(−)D period (38 ± 3 to 58 ± 4 pg/mL). Furthermore, they decreased to the baseline levels after switching back to A(+)D period (Figure 5B and C). Likewise, in the patients with int-PTH < 60 pg/mL, serum levels of BAP were significantly (*P* < 0.05) increased, from 12 ± 1 to 16 ± 2 U/L after switching for A(+)D period to the A(−)D period (Figure 5C), although there were no significant differences in serum BAP levels in the patients with 60≤ int-PTH <180 pg/mL or int-PTH ≥180 pg/mL during the study period (Figure 5A and C).

## Discussion

In a single session study, we compared the changes of t-calcium, i-calcium, and int-PTH during HD with A(−)D or A(+)D. We found significant increase in i-calcium, t-calcium and decrease in int-PTH after HD with A(+)D. In contrast, there was no significant difference in i-calcium and int-PTH levels after HD with A(−)D. These differences could be affected by the post-dialysis iCa levels. Despite the calcium concentrations of A(−)D and A(+)D being the same level (3.0 mEq/L), A(−)D contains a small amount of citrate as a buffer source. Because citrate is an effective chelator of i-calcium, HD with A(−)D containing citrate might decrease serum i-calcium levels. Therefore, Ca chelating effect of citrate as well as alkalizing effect of A(−)D might decrease iCa which affects intPTH.

In the cross-over study, we conducted a stratified analysis, according to the target int-PTH levels of 60–180 pg/mL, as recommended by the 2006 guidelines for the management of secondary hyperparathyroidism in chronic dialysis patients by JSDT [8]. In the patients with low int-PTH levels (< 60 pg/mL), we found significant increase in both int-PTH and BAP levels HD after A(−)D period, while it did not affect PTH levels in the patients with PTH levels >60 pg/ml. Thus, HD with A(−)D could contribute to the increase in PTH and BAP exclusively in the patients with low int-PTH levels.

### Calcium concentrations in dialysate and bone metabolism

Past studies have reported the impact of calcium concentrations in dialysates on bone mineral metabolism and int-PTH levels [3-5]. In the presence of low PTH levels, the use of a low calcium dialysate has been shown to increase circulating PTH and BAP levels in MHD patients [9,10]. However, with the excessive lowering of serum calcium during HD using a low calcium dialysate, calcium concentrations may be associated with hypotension and arrhythmias [11]. Furthermore, in patients with hyperparathyroidism and high bone turnover disease, the excessive use of a low calcium concentration dialysate may further worsen their conditions [12].

There were no significant differences in pre-dialysis t-calcium and i-calcium levels during the cross-over study. On the other hand, in the single session study, we found significant increases in i-calcium and t-calcium and decreases in int-PTH levels after HD with A(+)D, while there were no significant differences in i-calcium or int-PTH between pre- and post-HD levels with A(−)D in the single session study. From these results, we presumed that citrate in the dialysate could chelate i-calcium during HD sessions with A(−)D. Also from the results of this study, we concluded that not only the decrease in the calcium concentration of dialysate but also the chelation of i-calcium by citrate in dialysate might affect int-PTH levels in patients undergoing MHD.

### Metabolic acidosis and bone metabolism

The bone diseases that accompany CKD are due to alterations in PTH levels and vitamin D levels. However, a substantial quantity of data has been accrued to implicate chronic metabolic acidosis as an additional important factor [13]. In our cross-over study, although there was no significant difference in int-PTH or BAP during the A(−)D and A(+)D periods in the patients with normal or high PTH levels, we found a significant increase in int-PTH and BAP levels during the A(−)D period exclusively in the patients with low PTH levels. This result is in agreement with the previous study [7], which was performed on 21 MHD patients with metabolic acidosis (serum HCO_3_^-^ levels: 15–16.8 mmol/L). The amelioration of metabolic acidosis by raising the dialysate base HCO_3_^-^ concentration was found to the rise in PTH, reduce bone resorption in the patients with low PTH levels, on the other hand, improve bone formation in the patients with high PTH levels. Furthermore, Krieger et al. reported, from an in vitro study, a direct, suppressive effect of metabolic acidosis on osteoblastic-induced collagen synthesis, which decreased by 15% after exposure to an acidic milieu [14]. We did not know the exact mechanism why in crossover study PTH level as well as BAP increased exclusively in patients with low-PTH level. From these reports and our results, the optimal correction of metabolic acidosis might affect int-PTH and BAP levels in the patients with low PTH levels. On the other hand, there was no significant difference in int-PTH or BAP levels, in either the A(−)D period or A(+)D periods, in the patients with high PTH levels. Graham K. A. et al. [15] reported that, after the correction of metabolic acidosis in MHD patients with hyperparathyroidism, parathyroid hormone secretion was suppressed by increasing the sensitivity of the parathyroid glands to ionized calcium. Furthermore, sensitivity to vitamin D treatment in renal osteodystrophy is affected by metabolic acidosis, which induces resistance to vitamin D compounds and calcimimetics [16].

It had been reported that metabolic acidosis triggers an increase in bone resorption and a decrease in bone formation in parathyroidectomized rats [17]. Furthermore, bone metabolism is affected by pH modulation of calcium-sensing receptor (CaR) activation. Quinn S. J et al. reported that CaR can sense changes in pH. They showed that changes in extracellular pH produced changes in intracellular calcium levels, a key intracellular signal which mediates the inhibition of PTH secretion by CaR activation [18]. From these reports, we presumed that the optimal correction of metabolic acidosis might improve the response to vitamin D compounds and suppress the increase of int-PTH levels during the A(−)D period in the patients with high PTH levels.

### Inflammatory condition and bone metabolism

Acetate induces the expression of pro-inflammatory cytokines in monocytes and polymorphonuclear neutrophil leukocytes, which may be responsible for chronic inflammation in HD patients [19]. The removal of acetate from the dialysates and the purification of the dialysates would lead to a possible attenuation of chronic inflammatory conditions in MHD patients. Recently, Eleftheriadis T et al. demonstrated the negative correlation between serum IL-6 levels and bone turnover osteocalcin or the beta-isomerised C-terminal cross-linked peptide of collagen type I, which suggests a relationship between bone turnover and chronic inflammation in MHD patients [20]. These reports indicate that the attenuation of a chronic inflammatory condition may improve low bone turnover in MHD patients. Although, this study did not compare the inflammatory conditions of A(−)D and A(+)D, the improvement in bone turnover found in this study could be caused by the attenuation of the chronic inflammatory condition by A(−)D.

#### Limitations

The primary limitations of this study are the small patient population, the lack of values for pharmacological parameters such as the blood levels for citrate and acetate, and our inability to specify a single type of dialysis membrane, the glucose concentration or the pH in the dialysate.

## Conclusion

Although further studies are needed, the mechanisms involved in increased int-PTH and BAP levels in patients with low int-PTH levels using A(−)D are associated with the adsorption of i-calcium, improvements in metabolic acidosis and with nutritional status. Our data demonstrate that HD with A(−)D could increase int-PTH and BAP levels in MHD patients, especially in the patients with low PTH levels that could be associated with low turnover bone disease.

## Competing interests

The authors declare that they have no competing interests.

## Authors’ contributions

TK and MF participated in the design of the study. MY and AK performed the statistical analysis. YO and YH helped to draft the manuscript. AM and TN conceived of the study, and participated in its design of the study. All authors read and approved the final manuscript.

## Pre-publication history

The pre-publication history for this paper can be accessed here:

http://www.biomedcentral.com/1471-2369/14/18/prepub
